# Urinary Exosomal Tissue TIMP and Angiopoietin-1 Are Preoperative Novel Biomarkers of Well-Differentiated Thyroid Cancer

**DOI:** 10.3390/biomedicines11010024

**Published:** 2022-12-22

**Authors:** Chih-Yuan Wang, Shyang-Rong Shih, Kuen-Yuan Chen, Pei-Jie Huang

**Affiliations:** 1 Department of Internal Medicine, National Taiwan University Hospital, College of Medicine, National Taiwan University, Taipei 100225, Taiwan; 2 Department of Surgery, National Taiwan University Hospital, College of Medicine, National Taiwan University, Taipei 100225, Taiwan

**Keywords:** thyroid cancer, exosome, urine, angiopoietin-1, TIMP (tissue inhibitor of metalloproteinase)

## Abstract

Finding non-invasive and sensitive biomarkers for early screening of high-risk patients remains important in clinical practice. A higher concentration of urine exosomal thyroglobulin protein was found in late-stage patients with thyroid carcinoma compared to those with early stage in our previous study. This prospective study aims to find new prognostic biomarkers before surgery for decision-making with this platform. We enrolled patients newly diagnosed with papillary and follicular cancer from 2017 to 2018. Preoperative urine samples were collected and the exosomal proteins were analyzed. The association of the concentration of urine exosomal proteins with lymph node metastasis and MACIS score (metastasis, age, completeness of resection, invasion, and size) was analyzed with multiple logistic regression. In total, 21 patients were included, with a mean age of 51.29 ± 10.29 years and a majority of female patients (85.71%). The concentration of urine exosomal TIMP (tissue inhibitor of metalloproteinase) was significantly higher in patients with lymph node metastasis (*p* = 0.01). Multiple logistic regression analysis showed association of urine exosomal TIMP (adjusted odds ratio (aOR): 3.09, 95% confidence interval (CI): 0.99–9.6, *p* = 0.052), angiopoietin-1 (aOR: 2.24, 95% CI: 0.97–5.15, *p* = 0.058) with lymph node metastasis. However, no association was noted between MACIS score and various urine exosomal protein candidates. Preoperative urine exosomal data could suggest certain peptides having the potential as prognostic indicators for screening patients with high-risk before surgery. Further study with a large cohort and long follow-up is needed to identify the application of urine exosomal proteins on prognostic prediction.

## 1. Introduction

Thyroid cancer is a low-grade endocrine malignancy of high prevalence [1]. Although the prognosis is mostly good, the recurrence rate can be around 30% during the follow-up period [2]. Gender difference is an important feature of thyroid cancer. Usually, the ratio of thyroid cancer between men and women is around 5:1 [2]. Surgical intervention with radioactive iodine ablation usually plays a pivotal role initially. Therefore, it is important to find a life-long and non-invasive biomarker which can screen high-risk patients in the early stage or track the progression of recurrence annually after thyroidectomy [3], but without interference of serum anti-thyroglobulin antibody, as well as the expensive recombinant human TSH intervention or discomfort during withdrawal of thyroid hormone. We need a new concept to find certain biomarkers via a non-invasive and reproductive pathway. Exosomes are extracellular vesicles with a size range from 40 to 160 nm in diameter with an endosomal origin [4]. Exosomes derived from cancer cells have a strong capacity to modify both local and distant microenvironments [5]. Because exosomes can be detected in bodily fluids such as blood, urine, saliva, and cerebrospinal fluid, they might represent ideal non-invasive or less-invasive biomarkers for cancer diagnosis. The nanotechnology/nanomaterials aspect of exosomes will correlate with their interactions with intracellular organelles, which might generate probable therapeutic predictors or targets. Such diverse and interacted materials in exosomes include ribosomal nucleic acids, DNA, and peptides, with various cellular origin, and exosomes will provide a nano-window to observe and forecast cellular changes, such as cancer cell metastasis [4,5].

In fact, molecules enveloped in urine exosomes could be biologically starting ones with higher purity. Peptides will be preserved against degradation. The applications of exosomal molecules could include their use as biomarkers for disease diagnosis, prognosis, and efficacy of treatment. Our previous study showed that expression of urinary exosomal (UEx) thyroglobulin protein was significantly higher in patients with T3 stage or lymph node metastasis than those with T1–2 stage, suggesting UEx thyroglobulin expression correlated with the severity of thyroid cancer progression. Therefore, UEx thyroglobulin could act as a potentially biological marker for screening post-operative recurrence in high-risk patients [6]. In addition, Angiopoietin-1 in angiogenesis and tissue inhibitor of metalloproteinase (TIMP, endogenous inhibitors of matrix metalloproteinases) were also reported with cancer aggressiveness [4]. Based on the finding, this pilot study aims to use this platform to identify other prognostic biomarkers before surgery for decision-making and aggressive follow-up.

## 2. Materials and Methods

### 2.1. Patients

This prospective study enrolled patients with thyroid carcinoma in our hospital from 2017 to 2018. Inclusion criteria were new diagnosis with papillary thyroid carcinoma and follicular carcinoma. This study was approved by the ethics committees of the Institutional Review Board of National Taiwan University Hospital (201711082RINA) and was carried out following the approved guidelines. Informed consent was obtained from all participants in accordance with relevant guidelines and regulations. We collected the urine samples before and after one day of thyroidectomy, post-operatively at three and six months (four collections for every patient), and they received further follow-up for the next consecutive three years.

Patients’ urine samples were collected before operation or chemo-radiotherapy. Urine exosomes of different proteins were analyzed. Patients’ demography, serum thyroglobulin test, and clinical parameters were obtained from medical records. Outcome indicators are MACIS score and lymph node metastasis. MACIS score ≥7 is considered as a predictor for poor prognosis. MACIS score calculator (MACIS = 3.1 x (at age less than or equal to 39 years) or 0.08 x age (at age greater than or equal to 40 years) + 0.3 x tumor size (centimeters) + 1 (with incompletely thyroidectomy) + 1 (with locally invasive) + 3 (with distant metastases)) was provided by American Thyroid Association (accessed on 10 December 2022; https://www.thyroid.org/professionals/calculators/thyroid-cancer-staging-calculator/). The higher MACIS score, the more aggressive the tumor behavior and the poorer the prognosis. The association between outcomes and exosome expression was analyzed.

### 2.2. Exosome Collection

Individualized urinary exosome precipitation was carried out. A fresh human urinary sample of 200 mL was collected for exosome precipitation, centrifuged at 3000 g for 15 min at 4 °C to remove cells and debris, and 10,000 g for 30 min at 4 °C to remove microvesicles. We concentrated 200 mL urinary samples to 5–10 mL using Amicon^®^ Ultra 15-centrifugal filters, 100K (Millipore). Exo Quick-TC was used to isolate urinary exosomes (SBI). Supernatants were transferred to new tubes and stored at −80 °C with complete TM, EDTA-free Protease Inhibitor Cocktail (Roche). The suspended exosomes were then pelleted in lysis buffer (7 M urea, 2 M thiourea, 4 percent CHAPS). Exosome protein samples were kept frozen at 80 °C until they were analyzed using a multiple reaction monitor (MRM).

### 2.3. Tryptic Digestion

Three volumes of cold methanol at −20 °C were used to precipitate the urinary exosome samples, which were then centrifuged at 10,000 g for 10 min. After that, the pellet was suspended in lysis buffer (4 M urea, 25 mM ammonium bicarbonate, pH 8.5). The denatured samples were reduced with 200 mM dithiothreitol at room temperature for 1 h before being alkylated in the dark with 200 mM iodoacetamide. The remaining iodoacetamide was quenched with 200 mM DTT and incubated at room temperature for 20 min. Samples were treated with modified sequencing-grade trypsin (Promega, Madison, WI, USA). Digestion lasted 16 h at 37 °C.

### 2.4. MRM Q1/Q3 Ion Pair Selection Using Direct Infusion

Synthetic standard peptides were diluted to 2 ug/mL in 0.1 percent formic acid for infusion with a syringe pump at a flow rate of 10 uL/min. ESI was used to analyze the infused peptide solutions on an AB SCIEX QTRAP 5500 mass spectrometer equipped with the Turbo V source and controlled by Analyst software 1.5. The MS analysis was done in positive ion mode with an ion spray voltage of 5500 V. Source temperature was set to 550 °C. Additional parameters of note are the nebulizer and drying gas flow at 60 and 45 psi, respectively. The Analyst software (version 1.5) was used to generate a list of all possible b- and y-series fragment ions for both 2+ and 3+ precursor ion-charge state spanning m/z range from 100 to 1000. MRM scans for optimization of MRM Q1/Q3 ion pairs were conducted with both Q1 and Q3 set to unit resolution (0.7 Da FWHM), while the collision energy (CE) was ramped from 5 to 55 V in 1-V increments, with dwell time of 150 ms for each transition. On a per-peptide basis, the four transitions that produced the strongest signals were chosen from these data. Next, the three transitions producing the most abundant signals which were free of signal interferences were selected from these four transitions. 

### 2.5. LC-MRM/MS Analysis of Urinary Exosomes Digests

Using an Agilent 1260 Infinity HPLC system, 10 uL of urine digest samples were directly injected onto a reverse-phase analytical column (100 mm × 2.1 mm i.d., 2.7 um, Agilent Poroshell 120 EC-C18) that was kept at room temperature. Sample separations were achieved with a flow rate of 300 uL/min and a gradient of 3–90 percent mobile phase B over a total run time of 30 min. Mobile phase A was 0.1 percent *v*/*v* formic acid, and mobile phase B was ACN/0.1 percent formic acid. The gradient method is composed of multiple linear gradients as follows (time:%B): 0.1 min, 10% B; 3.5 min, 11% B; 6.5 min, 20% B; 7 min, 21% B; 7.5 min, 22% B; 12.5 min, 22.5% B; 17 min, 25% B; 20 min, 30% B; 22.5 min, 42% B; 23.5 min, 90% B; 27 min, 3% B; 30 min, 3% B. n AB SCIEX QTRAP 5500 with a Turbo V ionization source, controlled by Analyst software, was used for all LC-MRM/MS sample analyses. The following parameters were used in all acquisition methods: a 5500 V ion spray voltage, a nebulizer and drying gas flow of 60 and 45 psi, respectively, a source temperature of 550 °C, and Q1 and Q3 set to unit resolution (0.7 FWHM).

MRM acquisition methods were initially composed of four ion pairs per peptide during the determination of high-signal producing interference-free transitions and LC method development. The final analytical method was composed of one verified quantifier ion pair per peptide, and was presented as a demonstration of a high-throughput fast 30-min method, which had been checked for common urine interferences. However, the urine analysis of the samples was performed with acquisition methods containing three verified ion-pair transitions per target peptide in order to ensure the detection of any minor sample-specific signals which might occur. MRM acquisition methods were constructed with fragment ion-specific tuned CE voltages and retention time constraints.

### 2.6. MRM Data Analysis

All MRM data were processed using AB SCIEX Analyst software (version 1.5) with the Integrator algorithm for peak integration set with default values. All integrated peaks were manually inspected to ensure correct peak detection and accurate integration. Linear regression of all calibration curves was performed using a standard 1/x2 (x = concentration) weighting option to aid in covering a wide dynamic range. The concentration of each peptide target was calculated based on the observed response and the experimentally determined linear regression equation from the standard curve. The calculated concentration is reported in μM of urine and can be defined in ng/mL under the basis of the weight of the entire processed protein.

### 2.7. Thyroglobulin and Anti-Thyroglobulin Antibody

Thyroglobulin survey is determined by IMMULITE 2000 Thyroglobulin, which is solid-phase, chemiluminescent immunometric assay. Analytical sensitivity is 0.2 ng/mL, (Siemens, Erlangen, Germany). Anti-thyroglobulin antibody survey is determined by ARCHITECT Anti-Tg assay, which is a two-step immunoassay for quantitative determination of thyroglobulin autoantibodies in human serum. The sensitivity is designed having a limit of detection of ≤1.0 IU/mL (Abbott Laboratory, Abbott, Abbott Park, IL 60064, USA).

### 2.8. Reagents and Chemicals

All reagents were ACS grade or higher. All solvents used, including water, were LC/MS grade.

### 2.9. Statistical Analysis

Continuous data with normal distribution were presented as mean ± standard deviation, and continuous data without normal distribution are presented as median (interquartile: 25th–75th percentile, IQR). We respectively conducted Student’s *t*-test and Wilcoxon rank sum test to test the continuous data with normal distribution and without normal distribution to explore differences between preoperative exome expression and outcome. Categorical data are presented as *n* (%) and the difference between demography and outcome was evaluated by chi square test or Fisher’s exact test, as appropriate. Logistic regression model was used to estimate the odds ratio (OR) and 95% confidence interval (CI) for the relationship between pre-op exome expression and outcome. All statistics are two-sided and performed with SAS statistical software (version 9.4, Cary, NC, USA).

## 3. Results

### Characteristics of Study Population

Table 1 presents the baseline characteristics of participants. In total, 21 participants were included, with a median age of 49 years, and a majority (85.71%) were female. Furthermore, 19.05% and 23.81% of them suffered from hypertension and type 2 diabetes, respectively. The median MACIS score was 6.24 (IQR = 1.21, Q1—Q3: 5.64—6.85), with five patients ≥ 7 (23.81%), who were at high risk of recurrence. Eight (38.10%) patients had lymph node metastasis.

**Figure 1:**
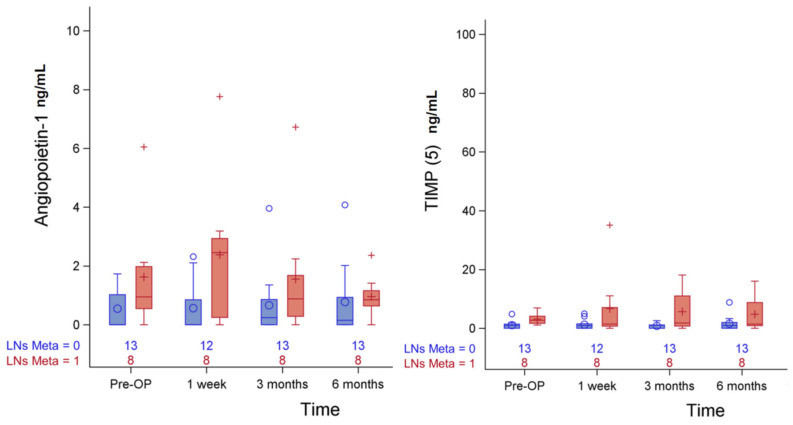
Serial change of urinary exosomal angiopoietin-1 and TIMP (5) in LNs Metas: before and after operation.

Usually, lymph nodes metastasis might suggest disease progression in well-differentiated thyroid cancer. Thus, it will be important to find certain earlier predictors for such metastasis or distant metastasis. The association between preoperative exosome protein level and lymph node metastasis is summarized in Table 2. Compared to patients with lymph node metastasis, those without lymph node metastasis were in TNM stage I (27.78% vs. 72.22%, *p* = 0.04), ≤1 time I-131 treatment (62.5% vs. 100%, *p* = 0.08), and a higher I-131 cumulative dose (45 (30–140) vs. 30 (0–30), *p* = 0.02). Higher cumulative dose of radioactive iodine revealed higher risk of lymph nodes metastasis. The serial changes with statistical significance or trend of angiopoietin-1and TIPM (5) in lymph node metastasis before and after operation are shown in Figure 1.

**Table 2:** The association between pre-operative expression of exosomal peptides and metastasis of lymph nodes.

Variable	LNs Metas		*p*-Value
	Yes (*n* = 8)	No (*n* = 13)	
Pre-op exosome expression			
UEx Thyroglobulin	1.08 (0.67–1.62)	2.21 (0.91–4.13)	0.33 ^b^
UEx Galectin-3	0.69 (0.57–2.39)	1.28 (0.72–2.28)	0.46 ^b^
UEx Transketolase	1.64 (0.45–13.36)	0.51 (0.19–1.27)	0.14 ^b^
UEx Calprotectin A8	10.10 (1.42–27.68)	42.00 (3.05–71.60)	0.17 ^b^
UEx Calprotectin A9(2) *	38.28 (17.24–252.80)	22.08 (4.88–67.58)	0.49 ^b^
UEx Calprotectin A9(13) *	19.68 (7.54–87.20)	5.20 (5.12–16.08)	0.14 ^b^
UEx Annexin-2	55.20 (25.60–165.60)	40.81 (19.90–119.59)	0.49 ^b^
UEx Afamin	0.60 (0.25–0.80)	0.31 (0.00–0.70)	0.58 ^b^
UEx Angiopoietin-1	0.95 (0.55–1.98)	0.00 (0.00–1.02)	0.09 ^b^
UEx Keratin-19	0.79 (0.56–1.45)	0.69 (0.53–1.54)	1.00 ^b^
UEx TIMP (5) *	2.73 (1.67–4.11)	0.95 (0.00–1.42)	0.01 ^b^
UEx TIMP (14) *	1.63 (0.33–6.38)	0.68 (0.19–1.26)	0.31 ^b^
UEx Keratin 8 (8) *	0.48 (0.23–1.07)	0.42 (0.23–0.80)	0.61 ^b^
UEx Keratin 8 (17) *	1.34 ± 0.92	0.94 ± 0.63	0.25 ^a^
Demography			
Gender			0.79 ^c^
Male	1 (33.33%)	2 (66.67%)	
Female	7 (38.89%)	11 (61.11%)	
Age (Year)	49.75 ± 13.77	52.23 ± 7.95	0.60 ^a^
Hypertension			0.62 ^c^
Yes	2 (50.00%)	2 (50.00%)	
No	6 (35.29%)	11 (64.71%)	
Diabetes (type 2)			1.00 ^c^
Yes	2 (40.00%)	3 (60.00%)	
No	6 (37.50%)	10 (62.50%)	
Clinical parameters			
TNM stage			0.04 ^c^
I	5 (27.78%)	13 (72.22%)	
II	3 (100.00%)	0 (0.00%)	
Tumor size (cm)	1.30 (0.65–1.60)	1.4 (0.9–1.7)	0.69 ^b^
Tumor multifocality			0.13 ^c^
Yes	2 (100.00%)	0 (0.00%)	
No	6 (31.58%)	13 (68.42%)	
Extrathyroid invasion	1 (16.67%)	5 (83.33%)	0.34 ^c^
I-131 treatment			0.08 ^c^
No	1 (16.67%)	5 (83.33%)	
1 time	4 (33.33%)	8 (66.67%)	
2 times	3 (100.00%)	0 (0.00%)	
I-131 cumulative dose	87.5 (30–300 mCi)	18.4 (0–30 mCi)	0.02 ^b^
ATA (IU/mL)			0.39 ^c^
<3	3 (27.27%)	8 (72.73%)	
≥3	5 (50.00%)	5 (50.00%)	
TPO Ab (IU/mL)			0.63 ^c^
<3	5 (33.33%)	10 (66.67%)	
≥3	3 (50.00%)	3 (50.00%)	
Post-operative TSH (IU/mL)	36.46 (1.25–78.85)	23.30 (0.96–111.00)	0.97 ^b^

ATA: anti-thyroglobulin antibody; TPO Ab: thyroid peroxidase antibody. Note: There was 1 patient missing of UExTg and UEx Galectin-3. Bold value denotes statistically significant, *p* < 0.05. ^a^ Using Student’s *t*-test. ^b^ Using Wilcoxon rank sum test. ^c^ Using Fisher’s exact test. * The numbers in brackets are peptide No. (Table 3).

**Table 3:** Peptide Standards List and Representative Protein (candidate peptide sequences).

Peptide No.	Sequence	Molecular Weight (Dalton)	Representative Protein
13	LGHPDTLNQGEFK	1455.59	CalprotectinA9
10	IIALDGDTK	945.08	Transketolase
15	GNDVAFHFNPR	1273.37	Galectin-3
9	AALEDTLAETEAR	1389.48	Keratin 19
4	QSSLILHGADFSTK	1503.68	Angiopoietin-1
6	IALDFQR	862	Galectin-3
5	FVGTPEVNQTTLYQR	1752.95	Tissue inhibitor of metalloproteinase
8	LSELEAALQR	1129.28	Keratin 8
1	ALNSIIDVYHK	1272.47	CalprotectinA8
14	GFQALGDAADIR	1233.35	Tissue inhibitor of metalloproteinase
12	VIFDANAPVAVR	1271.48	Thyroglobulin
17	ASLEAAIADAEQR	1344.44	Keratin 8
11	FLAVQSVISGR	1176.38	Thyroglobulin
16	AEDGSVIDYELIDQDAR	1908.99	Annexin II
3	FLVNLVK	832.05	Afamin
2	NIETIINTFHQYSVK	1807.04	CalprotectinA9
7	GVDEVTIVNILTNR	1542.75	Annexin II

**Figure 2:**
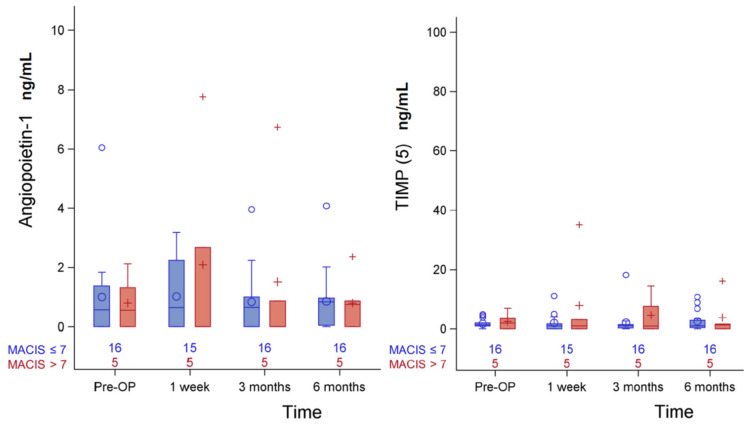
Serial change of urinary exosomal angiopoietin-1 and TIMP (5) in MACIS before and after operation.

Clinically, MACIS score has been used to evaluate the clinical course of well-differentiated thyroid cancers for years. However, is was no definite study investigating the correlation between certain predictors and MACIS score. The association between preoperative exosomal protein levels, and MACIS is summarized in Table 4. The patients with MACIS ≥ 7 were significantly older and had a higher proportion of type 2 diabetes in comparison with those with MACIS < 7 (both *p* = 0.004), and certain systemic diseases seemed to be a potential risk factor. The patients with TNM stage II (*p* = 0.008) and tumor multifocality (*p* = 0.048) had higher MACIS scores ≥7. Such results revealed different aspects in MACIS score from lymph nodes metastasis (Table 2), and urinary exosomal peptides could be the more precise biomarkers independent of MACIS score. The serial changes of angiopoietin-1 and TIPM (5) in clinical MACIS before and after operation is revealed in Figure 2, without statistical significance.

Table 5 presents the relationship between pre-op exosome expression and outcome based on multiple logistic regression. Since the enrolled patient number is limited, we used the factors (*p* < 0.1) related to MACIS and lymph node metastasis in the univariate analysis in Table 2 and Table 4 as the correction factors and excluded the factors that will cause complete separation of data points. For dealing with the quasi-complete separation of data points in multiple regression, we combined I-131 treatment from three classifications to two classifications (Yes/No). No significant association was found between the variables and MACIS ≥7 after adjusting with age or type 2 diabetes. However, UEx Angiopoietin-1 (aOR: 3.09, 95% CI: 0.99–9.60, *p* = 0.052) and UEx TIMP (5) (aOR: 2.24, 95% CI: 0.97–5.15, *p* = 0.058) have borderline significant positive effects on lymph node metastasis after adjusting for I-131 treatment (Yes/No) correction. Although our results were insufficient to achieve statistical significance, we have still found that Angiopoietin-1 and TIMP can be detected in the UEx proteins of patients before surgery, and there is a trend of positive correlation with lymphatic metastasis and higher odds ratio.

## 4. Discussion

Although the clinical outcome of well-differentiated thyroid cancer was usually good, such patients still need certain non-invasive predictive biomarkers for recurrence in life-long follow-up [1,2]. Our previous study showed that UEx thyroglobulin concentration correlated with severity of thyroid carcinoma progression, therefore had potential as a biomarker [6]. In this pilot study, we further showed association of preoperative UEx Angiotensin-1 and TIMP concentrations with lymph node metastasis, suggesting novel candidates for screening high-risk patients before surgery. A larger cohort with long-term follow-up is needed to clarify the feasibility of UEx proteins in tracking recurrence after thyroidectomy.

Developing more sensitive biomarkers for early screening of high-risk populations remains an issue in clinical oncology. Regression model analysis showed border associations between U-Ex angiopoietin-1, TIMP concentrations, and lymph node metastasis. Although thyroid carcinoma is a low-grade malignancy with good prognosis, lymph node metastasis is still associated with postoperative recurrence [7,8] and requires a more ambitious program of therapy and follow-up. In order to find biomarkers related to prognosis from exosomal proteins in urine, we adopted MRM analysis to provide an option with higher sensitivity and lower cost. We chose these peptides because these genes, such as Annexin 2, Calprotectin A8, Calprotectin A9, Keratin 19, Keratin 8, Angiopoietin-1, Tissue inhibitor of metalloproteinase (TIMP), and Afamin, are associated with cancer progression. Annexin 2 is believed to serve as a receptor for plasminogen, which functions to produce plasmin [9]. Recent research indicated Annexin 2 contributes to chemo-resistance and cancer progression in non-small-cell lung cancer and estrogen-receptor negative breast cancer [10,11], and there are drug development research studies about targeting Annexin 2 [12]. Calprotectin A8/A9 is a complex of the mammalian proteins S100A8 and S100A9 [13]. They are upregulated in HNSCC, AML, and tumor-infiltrating monocytes and macrophages to promote tumor migration and invasion [14,15,16,17,18]. Keratins 8 (KRT8) protein was reported to bind to annexin A2, a protein known to mediate apoptosis as well as the redox pathway in anaplastic thyroid carcinoma (ATC) [19]. Afamin is a protein encoded by the *AFM* gene in humans. It was reported that pretherapeutic plasma afamin concentration negatively correlated with severity of ovarian cancer, and an increased afamin concentration after chemotherapy was associated with increased overall survival [20]. Recent research indicated that Angiopoietin-1 played a role in lymph node metastasis and invasiveness of papillary thyroid carcinoma [21,22] TIMPs, which comprise a family of four protease inhibitors: TIMP1, TIMP2, TIMP3, and TIMP4 [23]. They were reported to participate in thyroid cancer progression [24,25].

Clinically, MACIS is commonly used for prognostic scoring system, and score ≥7 is regarded as a high risk in postoperative recurrence. However, no association was found between MACIS score and UEx proteins in the current study. So far, all included patients are in good disease control with a follow-up around 24 months. Given that both papillary and follicular thyroid carcinomas are low-grade and slow-developing [7], a follow-up longer than 5 years is needed to identify the application of UEx proteins in long term monitoring recurrence and residual tumor metastasis.

## 5. Limitations

We prepared fundamental technique and procedures via thyroid cancer cell line study [6]. Choosing serum or urinary samples was a very difficult decision; however, we finally made the decision to use urinary samples due to their reproducibility and non-invasiveness. We used the Urinary Exosome Protein Database as standard content (healthy donors) to establish our experimental and clinical hypothesis (accessed on 10 December 2022; https://esbl.nhlbi.nih.gov/UrinaryExosomes/). We have been trying to develop a new concept of cancer biomarker for more than 10 years. In fact, there were more studies providing solid evidence for urinary biomarkers in cancer fingerprints [26,27]. The research of our experiments will be rational [6]. However, this prospective study has several limitations. First, the sample size is small. Second, we did not clarify the feasibility of UEx proteins on detecting recurrence after thyroidectomy and I-131 ablation in our study scope. A study with larger cohort and long-term follow-up is ongoing to examine the application of UEx proteins on early detecting recurrence in annual tracking.

## 6. Conclusions

Since the growing trend of thyroid cancer was noted all over the world, it is very important to find certain non-invasive, convenient, and reproductive biomarkers to monitor the post-operative status lifelong. This pilot study was the first study to examine these onco-proteins in urinary exosomes with no interventional procedure annually. This non-invasive testing method will prominently improve the willingness of patients to return for consultation and improve the accuracy of diagnosis, and adopt more active treatment strategies for patients with metastases. Further studies are now ongoing and more results will be available in the near future.

## Figures and Tables

**Table 1:** Characteristic of baseline demography of enrolled patients.

Variable	Total
	(*n* = 21)
Female/Male	18/3 (85.71/14.29%)
Age (year)	51.29 ± 10.29
Hypertension	4 (19.05%)
Diabetes (type 2)	5 (23.81%)
TNM stage	
I	18 (85.71%)
II	3 (14.29%)
Tumor size (cm)	1.40 (0.9–1.6)
Tumor multifocality	2 (9.52%)
Extrathyroid invasion	6 (28.57%)
I-131 treatment	
No	6 (28.57%)
1 time	12 (57.14%)
2 times	3 (14.29%)
I-131 cumulative dose (mCi)	(0–300)
30	11 (52.38%)
60	1 (0.47%)
120	1 (0.47%)
130	1 (0.47%)
300	1 (0.47%)
ATA, ≥3 (IU/mL)	10 (47.62%)
TPO Ab, ≥3 (IU/mL)	6 (28.57)
Post-operative TSH (IU/mL)	23.30 (1.00–82.30)
MACIS	6.24 (5.64–6.85)
≥7	5 (23.81%)
LNs Metastasis	8 (38.10%)

ATA: anti-thyroglobulin antibody; TPO Ab: thyroid peroxidase antibody; Categorical data are presented as *n* (%); Continuous data with unnormal distribution are presented as median (IQR; interquartile).

**Table 4:** The association between pre-operative expression of exosomal peptides and MACIS (metastasis, age, completeness of resection, invasion, and size).

Variable	MACIS		*p*-Value
	<7 (*n* = 16)	≥7 (*n* = 5)	
Pre-op exosome expression			
UEx Thyroglobulin	1.35 (0.62–3.91)	1.87 (1.18–1.88)	0.73 ^b^
UEx Galectin-3	0.76 (0.59–1.55)	1.90 (1.60–2.84)	0.12 ^b^
UEx Transketolase	0.52 (0.27–2.17)	1.62 (0.95–23.84)	0.30 ^b^
UEx Calprotectin A8	24.10 (1.42–61.80)	37.81 (17.56–62.35)	0.30 ^b^
UEx Calprotectin A9(2) *	26.31 (5.96–70.39)	31.77 (30.03–241.96)	0.39 ^b^
UEx Calprotectin A9(13) *	5.55 (5.14–62.39)	17.07 (9.46–22.29)	0.54 ^b^
UEx Annexin-2	43.81 (20.47–194.20)	45.60 (28.48–58.62)	0.84 ^b^
UEx Afamin	0.60 (0.00–0.81)	0.00 (0.00–0.49)	0.35 ^b^
UEx Angiopoietin-1	0.57 (0.00–1.38)	0.56 (0.00–1.32)	1.00 ^b^
UEx Keratin-19	0.79 (0.53–1.39)	0.69 (0.57–1.67)	1.00 ^b^
UEx TIMP (5) *	1.31 (0.94–1.95)	1.93 (0.00–3.49)	0.84 ^b^
UEx TIMP (14) *	0.68 (0.21–1.20)	4.48 (1.48–8.28)	0.12 ^b^
UEx Keratin 8 (8) *	0.45 (0.34–0.67)	0.23 (0.11–1.60)	0.74 ^b^
UEx Keratin 8 (17) *	0.99 ± 0.64	1.40 ± 1.09	0.31 ^a^
Demography			
Gender			1.00 ^c^
Male	14 (77.78%)	4 (22.22%)	
Female	2 (66.67%)	1 (33.33%)	
Age	47.94 ± 7.38	62 ± 11.68	0.004 ^a^
Hypertension			0.23 ^c^
Yes	2 (50.00%)	2 (50.00%)	
No	14 (82.35%)	3 (17.65%)	
Diabetes (type 2)			0.004 ^c^
Yes	1 (20.00%)	4 (80.00%)	
No	15 (93.75%)	1 (6.25%)	
Clinical parameters			
TNM stage			0.008 ^c^
I	16 (88.89)	2 (11.11)	
II	0 (0.00)	3 (100.00)	
Tumor size	1.45 (0.85–1.65)	1.1 (1.0–1.5)	0.90 ^b^
Tumor multifocality			0.048 ^c^
Yes	0 (0.00%)	2 (100.00%)	
No	16 (84.21%)	3 (15.79%)	
Extrathyroid invasion	5 (83.33%)	1 (16.67)	0.85 ^c^
I-131 treatment			0.21 ^c^
No	5 (83.33%)	1 (16.67%)	
1 time	10 (83.33%)	2 (16.67%)	
2 times	1 (33.33%)	2 (66.67%)	
I-131 cumulative dose	30.6 (0–130 mCi)	84 (30–300 mCi)	0.32 ^b^
ATA (IU/mL)			0.45 ^c^
<3	9 (81.82%)	2 (18.18%)	
≥3	7 (70.00%)	3 (30.00%)	
TPO Ab (IU/mL)			0.45 ^c^
<3	12 (80.00%)	3 (20.00%)	
≥3	4 (66.67%)	2 (33.33%)	
Post-operative TSH (IU/mL)	4.55 (0.98–78.85)	71.28 (23.30–111.00)	0.49 ^b^

ATA: anti-thyroglobulin antibody; TPO Ab: thyroid peroxidase antibody; Note: There was 1 patient missing of UExTg and UEx Galectin-3. Bold value denotes statistically significant, *p* < 0.05. ^a^ Using Student’s *t*-test. ^b^ Using Wilcoxon rank sum test. ^c^ Using Fisher’s exact test. * The numbers in brackets are peptide No. (Table 3).

**Table 5:** The relationship between pre-operative expression of exosomal peptides based on multiple logistic regression.

Variable	MACIS ≥ 7 ^a^		LNs Metas ^b^	
	aOR (95% CI)	*p*-Value	aOR (95% CI)	*p*-Value
Pre-op exosome expression				
UEx Thyroglobulin	1.02 (0.57–1.81)	0.95	0.95 (0.77–1.16)	0.60
UEx Galectin-3	1.53 (0.48–4.88)	0.47	0.98 (0.64–1.49)	0.91
UEx Transketolase ^c^	NA	-	1.13 (0.92–1.38)	0.25
UEx Calprotectin A8	1.00 (0.99–1.01)	0.74	0.99 (0.98–1.01)	0.38
UEx Calprotectin A9(2) *	1.01 (0.99–1.02)	0.51	1.01 (1.00–1.02)	0.25
UEx Calprotectin A9(13) *	1.01 (0.97–1.05)	0.68	1.01 (0.99–1.03)	0.34
UEx Annexin-2	1.00 (0.99–1.01)	0.99	1.00 (1.00–1.00)	0.92
UEx Afamin	1.23 (0.28–5.49)	0.78	0.81 (0.40–1.67)	0.57
UEx Angiopoietin-1	1.41 (0.28–7.08)	0.68	3.09 (0.99–9.60)	0.052
UEx Keratin-19 ^c^	NA	-	0.85 (0.38–1.89)	0.70
UEx TIMP (5) *	2.84 (0.47–17.16)	0.26	2.24 (0.97–5.15)	0.058
UEx TIMP (14) *	1.15 (0.86–1.52)	0.35	1.03 (0.90–1.18)	0.67
UEx Keratin 8 (8) *	1.16 (0.20–6.60)	0.87	0.90 (0.30–2.72)	0.85
UEx Keratin 8 (17) *	8.57 (0.28–260.23)	0.22	2.16 (0.56–8.38)	0.27

Note: There was 1 patient missing of UExTg and UEx Galectin-3. The models were separated conducted by different exosome expression. ^a^ Adjusted OR were adjusted for age and type 2 diabetes. ^b^ Adjusted OR were adjusted for I-131 treatment. ^c^ The sample size was small result in complete separation of data points detected. * The numbers in brackets are peptide No. (Table 3).

## Data Availability

The data supporting reported results were stored in our private office and computers, National Taiwan University Hospital.
